# Downregulation of the Complement Cascade In Vitro, in Mice and in Patients with Cardiovascular Disease by the BET Protein Inhibitor Apabetalone (RVX-208)

**DOI:** 10.1007/s12265-017-9755-z

**Published:** 2017-05-31

**Authors:** Sylwia Wasiak, Dean Gilham, Laura M. Tsujikawa, Christopher Halliday, Cyrus Calosing, Ravi Jahagirdar, Jan Johansson, Michael Sweeney, Norman C. Wong, Ewelina Kulikowski

**Affiliations:** 1 Resverlogix Corp., Suite 300, 4820 Richard Road SW, Calgary, AB T3E 6L1 Canada; 2 Resverlogix Inc., San Francisco, CA USA

**Keywords:** Epigenetics, Bromodomain, Bromodomain and extraterminal protein, Biomarker, Inflammation, Complement cascade, Innate immunity, Cardiovascular disease; major acute cardiac event

## Abstract

Apabetalone (RVX-208) is an epigenetic regulator developed to treat cardiovascular disease (CVD) that targets BET proteins. Through transcriptional regulation RVX-208 modulates pathways that underlie CVD including reverse cholesterol transport, vascular inflammation, coagulation, and complement. Using transcriptomics and proteomics we show that complement is one of the top pathways downregulated by RVX-208 in primary human hepatocytes (PHH) and in plasma from CVD patients. RVX-208 reduces basal and cytokine-driven expression of complement factors in PHH and in chimeric mice with humanized livers. Plasma proteomics of CVD patients shows that RVX-208 decreases complement proteins and regulators, including complement activators SAP and CRP. Circulating activated fragments C5a, C3b, and C5b-C6 are reduced by 51, 32, and 10%, respectively, indicating decreased activity of complement in patients. As complement components are linked to CVD and metabolic syndrome, including major acute cardiac events, modulating their levels and activity by RVX-208 may alleviate risks associated with these diseases.

**Electronic supplementary material:**

The online version of this article (doi:10.1007/s12265-017-9755-z) contains supplementary material, which is available to authorized users.

## Introduction

**Fig. 1:**
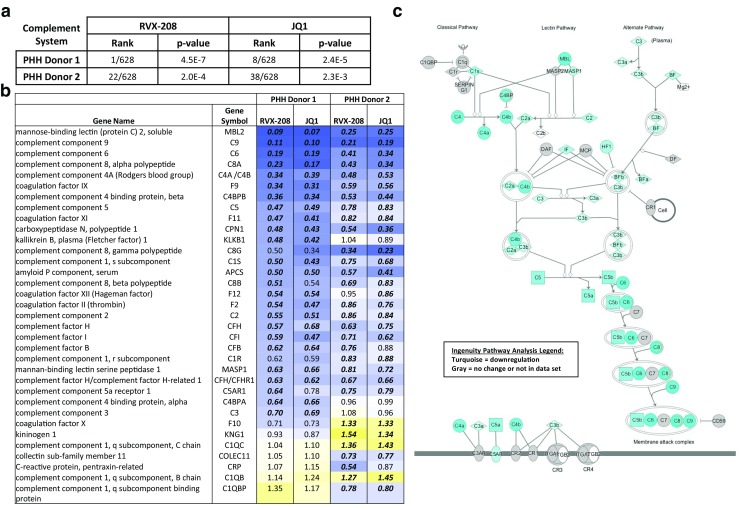
The complement system is the top downregulated canonical pathway in RVX-208-treated primary human hepatocytes. **a** Gene expression data was obtained by microarray analysis of triplicate samples of human primary hepatocytes from two donors (donor 1: child, donor 2: adult) treated with 30 μM RVX-208 for 48 h. Ranking of the complement canonical pathway was calculated with Ingenuity® Pathway Analysis (IPA®). **b** Complement gene expression changes relative to vehicle (1). *Blue* indicates downregulation, and *yellow* indicates upregulation. *Bold* indicates *p* value 0.05, Student’s *t* test. **c** Visualization of gene expression changes (IPA®) within the complement system induced by RVX-208 in hepatocytes from donor 1. *Turquoise* indicates downregulation

The complement system is an essential component of the innate immunity and a major trigger of host inflammatory responses. As such, it helps immune cells fight infections and maintains homeostasis by eliminating pathogens, immune complexes, debris, and apoptotic cells. The complement system is composed of more than 30 proteins, some of which are secreted into the bloodstream by hepatocytes, while others are associated with plasma membrane of many cell types. Most of circulating complement proteins participate in activation of the complement cascade, serving as enzymes, enzyme cofactors, or precursors of biologically active fragments (reviewed in [1]; see Fig. 1c).

Depending on the context the complement system can be activated through three distinct pathways: classical, lectin, and alternative, each leading to a common terminal C5-driven pathway (reviewed in [1]; see Fig. 1c). The classical pathway is activated by C1q which recognizes antigen-antibody complexes, microbial and apoptotic cells, and soluble pattern recognition receptors (PRRs) such as the C-reactive protein (CRP). In association with C1s and C1r, C1q propagates signaling through C4 and C2, leading to C3 cleavage and activation. The lectin pathway is activated by soluble PRR mannose-binding lectins (MBLs), ficolins, and collectins. Subsequently, activation of MBL-associated serine proteases (MASPs) leads to cleavage and activation of C3. Lastly, the alternative pathway involves a spontaneous cleavage of C3 in conjunction with factor B, factor D, and properdin, making C3 an integrator of all the signals that stimulate the complement cascade. Cleavage of C3 generates the C3b fragment which in turn cleaves and activates C5, the initiator of the terminal pathway. The cleavage fragment C5b associates with C6, C7, C8, and C9 to form C5b-9 on the membrane of target cells, leading to cell permeabilization and lysis. Soluble C5b-containing end products of complement activation are eliminated by clusterin and vitronectin to avoid unwanted association with non-target membranes. Cleavage of C3 and C5 not only promotes cell lysis but also contributes to inflammation and leukocyte recruitment through anaphylatoxic activity of the C3a and C5a fragments. These are inactivated by carboxypeptidase N that cleaves their C-terminal arginine residue to yield C3a-desArg and C5a-desArg. There are multiple proteins which downregulate complement activity, including regulators of the classical and lectin pathways C1 inhibitor and C4b-binding protein, and regulators of the alternative pathway Factor H and Factor I. Extensive cross talk between the coagulation and complement pathways also leads to activation of C3 and C5 via extrinsic proteases of the coagulation system (reviewed in [2]). Specifically, thrombin and activated coagulation factors IXa, Xa, XIa, and plasmin can directly cleave C3 and C5 to generate pro-inflammatory C3a and C5a. Factor XIIa has also been demonstrated to initiate classical pathway activation via cleavage of C1r. Conversely, activated complement fragments C3a, C5a, and C5b-C9 are known to enhance platelet activation and aggregation and promote thrombin generation on the platelet surface and fibrin formation, suggesting that complement activation may contribute to thrombus formation [3].

Complement expression and activity are tightly regulated to avoid immune dysregulation and host tissue damage. Both inefficient activation and overstimulation of complement can be detrimental for the host, and is associated with increased susceptibility to infectious diseases and non-infectious disorders with an autoimmune and chronic inflammatory component. Specifically, excessive complement expression and activity are implicated in several aspects of human cardiometabolic disease, including atherosclerosis, diabetes, metabolic syndrome, and acute coronary syndrome (reviewed in [4]).

Apabetalone (RVX-208) is an orally available small molecule developed for the treatment of cardiovascular disease (CVD) that targets epigenetic regulators bromodomain and extraterminal (BET) proteins BRD2, BRD3, and BRD4 [5]. Mechanistically, RVX-208 precludes interactions of BET proteins with acetylated lysines on histone tails that normally promote active transcription [5, 6]. This inhibition is mediated through binding of RVX-208 to bromodomains 1 and 2 of BET proteins [5, 6]. Of note, RVX-208 binds preferentially to BD2, which differentiates it from pan-BET inhibitors that target BD1 and BD2 with equal affinity [6]. RVX-208 is the first BET inhibitor (BETi) to enter human clinical trials for treatment of chronic disease, and analysis of its in vivo activity is ongoing [7–10]. A post hoc analysis of pooled data from phase IIb trials SUSTAIN and ASSURE showed that RVX-208 treatment moderately enhances the plasma lipid profile by increasing high-density lipoprotein cholesterol (HDLc) and ApoA-I while substantially lowering the incidence of major adverse cardiac events (MACE) in CVD patients [10]. These data prompted an investigation of beneficial effects of RVX-208 beyond the improvement of the lipid profile. Of note, BET inhibition by RVX-208 reduces atherosclerosis and expression of inflammatory markers in the ApoE mouse knockout model [11, 12]. In cultured primary human hepatocytes RVX-208 modulates expression of genes and pathways that underlie CVD, including reverse cholesterol transport, vascular inflammation, coagulation, and complement [10]. Here, using transcriptomics, real-time PCR, ELISA assays, and proteomics, we demonstrate that the complement cascade is broadly and coordinately downregulated by RVX-208 in primary human hepatocytes (PHH), in mice and in CVD patients. Our studies clearly indicate that RVX-208 impacts complement protein abundance and activation in vivo, and suggest that complement repression by RVX-208 may contribute to the decreased CVD risk and lower incidence of MACE observed in RVX-208 clinical trials.

## Methods

### Tissue Culture

Cryopreserved primary human hepatocytes (CellzDirect/Life Technologies) were plated and then overlaid with Matrigel™ as recommended by the supplier. Cells were treated with compounds dissolved in DMSO (0.1% final concentration) and/or cytokines for the indicated time in media recommended by the supplier supplemented with 10% fetal bovine serum (*v*/*v*). Huh-7 cells (JCRB Cell Bank) were introduced to 96-well plates (∼2.5 × 10^5^ per well) in 100 μL DMEM containing 10% (*v*/*v*) FBS, 100 U/mL penicillin, 100 μg/mL streptomycin, and 5 μg/mL plasmocin (all reagents from Life Technologies, except for the latter, which was obtained from InvivoGen). After 24 h, Huh-7 cells were treated with compounds dissolved in DMSO (0.1% final concentration) and/or cytokines for 72 h. In a pre-treatment protocol, 24 h after plating, cells were pre-treated with cytokines for 24 h, before addition of compounds for 48 h. Fresh media containing cytokines and compounds were added to cells 24 h prior to collection of cells and media.

### Gene Expression Microarrays

Cryopreserved primary human hepatocytes (CellzDirect™/ThermoFisher Scientific) were treated with DMSO (control) or RVX-208 at 30 μM for 48 h in triplicate. Total RNA was extracted with the mirVana™ kit (Ambion) and sent to Asuragen, Inc. (Austin, TX) for microarray analysis using Affymetrix Human Genome U133 Plus 2.0 Array. Triplicate data was averaged to calculate fold change in gene expression relative to DMSO-treated controls. Data were analyzed through the use of QIAGEN’s Ingenuity® Pathway Analysis software (IPA®, QIAGEN Redwood City, www.qiagen.com/ingenuity). Activation *z*-scores were calculated using IPA and represent the bias in gene regulation that predicts whether the pathway exists in an activated or inactivated state.

### Quantification of mRNA

Cells were harvested by mRNA Catcher PLUS Kit followed by real-time PCR using the RNA UltraSense™ One-Step qRT-PCR System (ThermoFisher Scientific). The level of the messenger RNA (mRNA) of interest was measured by TaqMan real-time PCR assays relative to the endogenous control cyclophilin A in a duplex reaction. Data were acquired using the ViiA-7 Real-Time PCR System (Applied Biosystems).

### ELISA

Media from treated cell cultures were collected over the final 24 h of the experiment, flashed frozen in liquid nitrogen, and stored at −80 °C. C3, C4, C5, and C9 proteins were detected with ELISA kits as per the manufacturer’s instructions (AssayPro). Abundance of proteins secreted by Huh-7 and HepG2 were normalized to cell number (quantified with CellTiter 96® AQ_ueous_ One Solution Cell Proliferation Assay (Promega)).

### Chimeric Mice with Humanized Liver

Urokinase-type plasminogen activator (uPA)/severe combined immunodeficient (SCID) mice with livers repopulated with human hepatocytes were generated as described previously [13] (PhoenixBio, Co., Ltd., Higashihiroshima, Japan). Protocols for the animal experiments were approved by the Laboratory Animal Ethics Committee at PhoenixBio Co., Ltd. (Resolution No. 0740). In vivo experiments were performed in a facility approved by the Office of Laboratory Animal Welfare (OLAW). Mice received RVX-208 at 150 mg/kg b.i.d. or vehicle by oral gavage for three consecutive days. Animals were anesthetized with isoflurane and sacrificed by cardiac puncture and exsanguination. Next, whole livers were harvested, rinsed in cold PBS, flash frozen in liquid nitrogen, and stored at −80 °C. Total RNA was extracted from chimeric livers with TRIzol® Reagent (Life Technologies) and was reverse transcribed with High-Capacity cDNA Reverse Transcription Kit (TermoFisher Scientific). TaqMan Gene Expression Master Mix (Applied Biosystems) was used to measure gene expression as above.

### SOMAscan™ Proteomic Analysis

The full design and rationale of the two studies, ASSERT (NCT01058018) and ASSURE (NCT01067820), has been published previously [8, 14]. The study protocol was approved by the institutional review board at each site. Patients provided written informed consent prior to study entry. The study conformed to the ethical principles contained in the Declaration of Helsinki. In both trials, patients received 200 mg RVX-208 daily. Major difference in the patient groups in ASSERT versus ASSURE was length of treatment (12 versus 26 weeks) and the severity of the coronary artery disease (CAD) (patients with documented CAD versus patients with coronary angiography for a clinical indication). Baseline and terminal plasma samples from ASSERT and ASSURE were analyzed using SOMAScan® proteomic technology that uses somamers as an affinity reagent (SomaLogic Inc., Boulder, CO). Plasma samples from 25 placebo and 30 RVX-208-treated patients from ASSERT and 47 samples each from the placebo and RVX-208 groups from ASSURE were analyzed for the presence of circulating proteins, including complement components and regulators. Changes in relative fluorescent units, which are directly proportional to the amount of target protein in the initial sample, were measured relative to baseline [15]. Shapiro-Wilk tests were used to determine data distribution. For normally distributed parameters, paired Student *t* tests were used to calculate statistical significance versus baseline, while for non-normally distributed parameters, Wilcoxon signed-rank tests were applied versus baseline. Mann-Whitney and Student *t* tests were used to compare median or mean change and percent change between RVX-208-treated patients and placebo depending on normality. Resulting data were analyzed through the use of QIAGEN’s Ingenuity® Pathway Analysis software (IPA®, QIAGEN Redwood City, www.qiagen.com/ingenuity).

## Results

### RVX-208 Downregulates Expression of Complement in Primary Human Hepatocytes, Huh-7 Cells, and Humanized Livers from Chimeric Mice

**Fig. 2:**
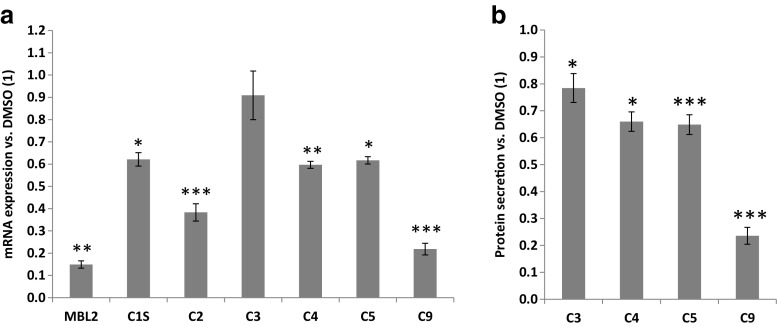
RVX-208 downregulates expression and secretion of key complement cascade components in primary human hepatocytes. Primary human hepatocytes from three donors were treated with 30 μM RVX-208 for 72 h. **a** Gene expression was analyzed by real-time PCR and expressed as fold change relative to vehicle (*1*). **b** Protein secretion was analyzed by ELISA, normalized to cell number, and expressed as fold change relative to vehicle (1). Standard deviation was calculated on technical triplicates. Representative data from one donor is shown (Student’s *t* test; *0.05> *p* >0.01; **0.01> *p* >0.001; ***p < 0.001)

Previously, RVX-208 was reported to downregulate the complement cascade in cultured PHH from a child donor [10]. Here, we have expanded our study of RVX-208 effects on complement genes by comparing the microarray data from RVX-208-treated PHH obtained from a child (donor 1) to PHH from an adult (donor 2). Analysis of the gene expression data using the Ingenuity® Pathway Analysis (IPA) highlighted the complement system as one of the top ranking canonical pathways affected by RVX-208 (1/628 for donor 1 and 22/628 for donor 2; Fig. 1a). A number of genes showed a differential RVX-208 response in those two donors (Fig. 2b). These differences are likely explained by dissimilarities in the donors age (3 versus 47 years old) and cause of death (motor vehicle accident versus stroke). Despite these distinct differences, many of the same genes central to the complement cascade were significantly downregulated in both donors, including genes within the classical, lectin, and alternative pathways (Fig. 1b, c). This widespread and coordinated downregulation not only affected complement cascade components but also complement activators, including soluble PRRs CRP, serum amyloid P (APCS), and collectin-11 (COLEC11), and coagulation cascade components factor II (thrombin), factor IX (F9), factor X (F10), factor XI (F11), factor XII (F12), and kallikrein B1 (KLKB1; Fig. 1b). Moreover, complement cascade inhibitors including factor I (CFI), factor H (CFH), C4-binding protein (C4BPA and C4BPB), and carboxypeptidase N (CPN) were downregulated (Fig. 1b). Of note, expression of the C5a receptor C5AR1 was also reduced (Fig. 1b).

To expand our understanding of BETi effects on the complement cascade, we assessed the effects of JQ1, a BET inhibitor with a chemical scaffold distinct from RVX-208, on PHH gene expression. Strikingly, JQ1 and RVX-208 showed the same pattern of complement gene repression between the two donors (Fig. 1b), consistent with on-target activity.

To validate the microarray data the downregulation of select complement genes that are central to the cascade, including MBL2, C1S, C2, C3, C4, C5, and C9, was confirmed by real-time PCR in three PHH donors (representative data shown in Fig. 2a). Downregulation of C3, C4, C5, and C9 mRNA was dose- and time-dependent, with maximal effects within 24 h of incubation with RVX-208 (Supplemental Fig. 1). Protein secretion of central complement pathway components C3, C4, C5, and C9 was also downregulated by RVX-208 (as measured by ELISA), similar to gene expression (Fig. 2b).

RVX-208 treatment also effectively decreased the complement pathway in the Huh-7 hepatocarcinoma cell line, where it suppressed MBL2, C1S, C3, C4, and C5 gene expression as measured by real-time PCR (C9 expression was not detectable) (Supplemental Fig. 2a). A dose-dependent downregulation of C3, C4, and C5 protein secretion was also observed by ELISA (Supplemental Fig. 2b). JQ1 also suppressed complement expression in Huh-7 cells and PHH, indicating that downregulation of complement transcription is a shared property of BET inhibitors (Supplemental Figs. 2 and 3, respectively).

**Fig. 3:**
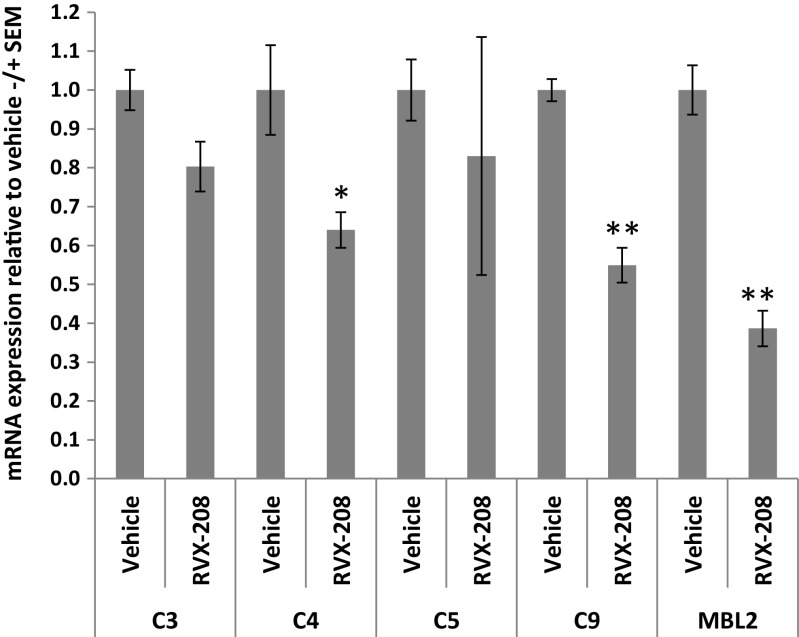
RVX-208 downregulates expression of complement components C4, C9, and MBL2 in chimeric mice with humanized livers. Mice were treated with 150 mg/kg b.i.d. RVX-208 or vehicle by oral gavage for three consecutive days, followed by chimeric liver human mRNA analysis. Standard error of the mean (SEM) was calculated on values obtained from three mice. Student’s *t* test; *0.05> *p* >0.01; **0.01> *p* >0.001

To assess effects of RVX-208 on complement gene expression in vivo, we used homozygous albumin enhancer/promoter-driven urokinase-type plasminogen activator/severe combined immunodeficient (uPA/SCID) mice with humanized livers [13]. Using this chimeric mouse model, replacement of mouse hepatocytes with PHH can reach 80–90%, allowing for study of human cells in vivo. Mice were treated with 150 mg/kg b.i.d. RVX-208 or vehicle by oral gavage for three consecutive days, followed by liver mRNA analysis. RVX-208 significantly reduced expression of human complement genes C4, C9, and MBL2 by 36, 45, and 61%, respectively (Fig. 3). Overall, data demonstrates that RVX-208 can downregulate complement gene transcription in human primary cells, in cell lines, and in humanized liver.

### RVX-208 Reduces Cytokine-Driven Expression of Complement in PHH and Hepatocarcinoma Cell Lines

**Fig. 4:**
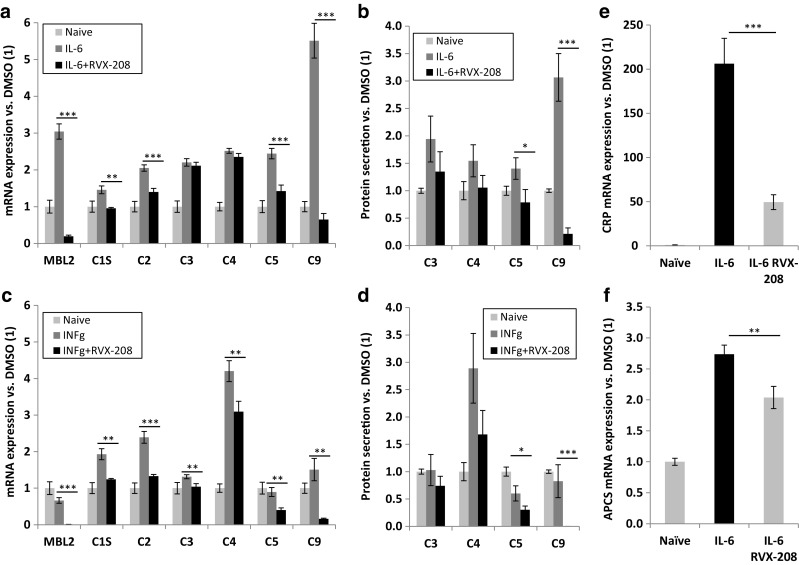
RVX-208 reduces inflammatory mRNA expression and protein secretion of complement factors in PHH. PHH were treated with 30 μM RVX-208 in combination with IL-6 (**a**, **b**) or interferon γ (**c**, **d**) (10 ng/mL) for a total of 72 h. Gene expression (**a**, **c**) was analyzed by real-time PCR and expressed fold change relative to naïve cells (*1*). Protein secretion (**b**, **d**) over the final 24 h of treatment was analyzed by ELISA, normalized to cell number, and expressed as fold change relative to naïve cells (1). CRP (**e**) and APCS (**f**) mRNA expression in naïve and IL-6 stimulated PHH was analyzed by real-time PCR. Standard deviation was calculated on technical triplicates. Representative data from three independent experiments is shown. Student’s *t* test; *0.05> *p* >0.01; **0.01> *p* >0.001; ***p < 0.001

Most complement proteins are synthesized by the liver and are classified as acute phase reactants, i.e., proteins of hepatic origin whose plasma concentrations increase following tissue injury or inflammation [16, 17]. These increases result from actions of various cytokines including interleukin-6 (IL-6), interleukin-1 (IL-1), tumor necrosis factor α (TNFα), and interferon γ (INFγ) [18–20]. BETi have well-established anti-inflammatory properties and have been reported to interfere with cytokine signaling pathways [12, 21–24]. Thus, we assessed here the effect of RVX-208 on cytokine-driven complement expression in PHH and hepatocarcinoma cells. IL-6, IL-1α, IL-1β, TNFα, and INFγ were applied to PHH and Huh-7 cells for 72 h. In PHH, TNFα and IL-1β produced limited induction of complement genes (data not shown). In contrast, both IL-6 and INFγ induced C1S, C2, and C4, while IL-6 (but not INFγ) induced MBL2, C3, C5, and C9 (Fig. 4a, c). Co-treatment with 30 μM RVX-208 reduced cytokine-induced complement gene expression (Fig. 4a, c). Protein secretion data for C3, C4, C5, and C9 mirrored effects of RVX-208 on gene expression (Fig. 4b, d). Inflammatory expression of complement cascade activators CRP and serum amyloid P (APCS) was also strongly downregulated by RVX-208 in PHH (Fig. 4e, f). In Huh-7 cells, RVX-208 strongly repressed cytokine-induced expression of MBL2, C1S, C2, C3, C4, and C5 (C9 was not detectable), often returning complement mRNA abundance back to baseline or below (Supplemental Fig. 4a). Under inflammatory conditions, secretion of C3, C4, and C5 was reduced by RVX-208 similar to mRNA (Supplemental Fig. 4b). RVX-208 was equally effective in PHH and Huh-7 cells when inflammatory expression was established with a 24-h cytokine pre-treatment prior to addition of the compound for 48 h (Supplemental Figs. 5 and 6, respectively), demonstrating that inflammatory gene expression can be reversed by the compound.

### RVX-208 Decreases the Abundance of Circulating Complement Proteins and Complement Activation Products in CVD Patients

To determine if RVX-208 can affect levels of circulating complement proteins in humans, the SOMAscan™ proteomic assay was performed on plasma from patients enrolled in ASSERT and ASSURE phase 2b clinical trials (Tables 1 and 2, respectively). SOMAscan® is a highly sensitive technology used to detect approximately 40 proteins and activated fragments that compose or regulate the complement cascade, making it the most complete complement proteome detection tool available. Comparison of data from placebo- versus RVX-208-treated patients from both trials showed downregulation of multiple cascade components (factor B, C2, C3, C4, C5, C6, C8, and C9), cascade inhibitors (factor I, CD55, C1INH, and vitronectin), and cascade activators (CRP, APCS, collectin-11, properdin), with a significant (*p* < 0.05) or trending towards significant (*p* < 0.1) *p* value. Moreover, a substantial reduction of cleaved complement fragments that reflect complement activation was observed, including C3b (32.3%, *p* = 0.06), C5a (51.4%, *p* = 0.0001), and C5b-C6 (10.4%, *p* = 0.002) (Tables 1 and 2). Coagulation factors did not show consistent changes between the two studies, which may be due to timing of blood sampling. No increased incidence of infestations or infections was detected in patients receiving RVX-208 in any clinical study (Supplemental Table 1).

**Table 1:** RVX-208 decreases the abundance of complement components and regulators in CVD patients from ASSERT IIb trial

Protein name	Gene symbol	Placebo	*p* value vs baseline	RVX-208 200 mg daily	*p* value vs baseline	Delta between treated and placebo	*p* value vs placebo
C-reactive protein	CRP	18.4	0.04	−24.3	0.2	−42.7	0.01
Complement C3	C3	−0.6	0.6	−13.9	0.1	−13.3	0.05
Vitronectin	VTN	4.5	0.2	−8.1	0.0	−12.6	0.02
Complement C4	C4A C4B	4.4	0.3	−5.7	0.1	−10.1	0.04
Coagulation factor X	F10	−2.6	0.06	2.7	0.3	5.3	0.03
Complement component C7	C7	−0.7	0.2	6.7	0.1	7.4	0.03
Complement C3b	C3	27.3	0.03	−5.0	0.3	−32.3	0.06
Properdin	CFP	8.1	0.4	−6.2	0.1	−14.3	0.10
Complement component C6	C6	−1.0	0.8	−8.6	0.03	−7.6	0.10
Complement factor I	CFI	2.1	0.7	−5.4	0.04	−7.5	0.05
Prothrombin	F2	0.5	0.6	-6.1	0.1	-6.6	0.06
Complement component C9	C9	−0.6	0.9	−5.7	0.1	−5.1	0.06

Protein abundance in plasma samples was determined using SOMAscan proteomic analysis and expressed as percent change between baseline and terminal visits in placebo- and RVX-208-treated groups. Differences between treated versus placebo greater than 5% with *p* < 0.05 (light gray) or 0.1> *p* >0.05 (dark gray) are shown

**Table 2:** RVX-208 decreases the abundance of complement components and regulators in CVD patients from ASSURE IIb trial

Protein name	Gene symbol	Placebo	*p* value vs baseline	RVX-208 200 mg daily	*p* value vs baseline	Delta between treated and placebo	*p* value vs placebo
C5a anaphylatoxin	C5	22.7	0.01	−28.7	0.0002	−51.4	0.0001
C-reactive protein CRP	CRP	-22.3	0.004	-43.6	0.0001	-21.3	0.02
Complement component C6	C6	0.9	0.9	−15.3	0.0001	−16.1	0.002
Collectin-11	COLEC11	15.4	0.1	0.7	0.5	−14.7	0.04
Complement component C8	C8A C8B C8G	1.9	0.3	−10.1	0.004	−12.0	0.01
Plasma protease C1 inhibitor	SerpinG1	3.4	0.3	−8.5	0.01	−11.9	0.01
Complement C2	C2	4.2	0.2	−6.7	0.001	−10.9	0.0002
Complement C5	C5	−0.9	0.4	−11.7	0.0001	−10.8	0.0001
Serum amyloid P-component	APCS	−2.1	0.1	−12.9	0.0001	−10.8	0.001
Complement C5b-C6 complex	C5 C6	−1.6	0.1	−12.0	0.0001	−10.4	0.002
Collectin-12	COLEC12	13.6	0.004	4.0	0.9	-9.6	0.02
Coagulation factor Xa	F10	7.4	0.002	−1.0	0.4	−8.5	0.005
Complement decay-accelerating factor	CD55	2.0	0.2	−6.3	0.03	−8.3	0.02
Coagulation factor X	F10	6.4	0.005	−0.2	0.5	−6.6	0.003
Complement factor B	CFB	−1.1	0.3	−6.8	0.001	−5.7	0.05
Thrombin	F2	−17.6	0.002	−3.0	1	14.6	0.02
Ficolin 1	FCN1	−19.2	0.0001	−2.8	0.5	16.4	0.04
Complement C1r subcomponent	C1R	−14.9	0.005	14.8	0.1	29.7	0.01

Protein abundance in plasma samples was determined using SOMAscan proteomic analysis and expressed as percent change between baseline and terminal visits in placebo- and RVX-208-treated groups. Differences between treated versus placebo greater than 5% with *p* < 0.05 (light gray) or 0.1> *p* >0.05 (dark gray) are shown

## Discussion

In this study we demonstrate a coordinated repressive effect of BET inhibitors RVX-208 and JQ1 on expression of multiple complement cascade components and regulators in primary human hepatocytes, hepatocarcinoma cells, mice with humanized liver, and human plasma samples. Elevated complement expression is linked to several chronic human diseases, including CVD, diabetes, metabolic syndrome, and acute coronary syndrome (reviewed in [4]). Thus, modulating levels of complement factors with RVX-208 may help alleviate risks associated with those diseases.

Most complement factors participate in the acute phase response and are upregulated in response to infection and tissue injury as well as during acute and chronic inflammation [20]. Those triggers stimulate immune cells to produce cytokines such as IL-6, IL-1, TNFα, and INF-γ which, in turn, can induce complement expression in hepatocytes [20, 25–28]. As shown here RVX-208 represses basal and cytokine-induced complement expression in both PHH and hepatocarcinoma cells. RVX-208 treatment is efficacious not only when co-applied with cytokines but also when inflammatory expression is induced prior to addition of the compound, suggesting that RVX-208 can interfere with pre-established inflammatory signaling in a therapeutic model. RVX-208 likely inhibits inflammatory expression by interfering with transcription factors that regulate responses to IL-1, IL-6, INFγ, and TNFα, including CAAT/enhancer-binding protein (C/EBP), NFκB, and signal transducers and activator of transcription (STATs), as shown for other BETi [12, 29–31]. Overall, our findings suggest that RVX-208 could lower secretion of complement proteins by hepatocytes during systemic acute or chronic inflammation. Moreover, since complement components have been shown to contribute to inflammatory and fibrotic liver diseases in humans and in mouse models, downregulating their abundance in liver tissue may prove beneficial for these conditions [32–35].

Proteomic analysis of plasma from RVX-208-treated patients showed a reduction in circulating levels of multiple complement components and regulators versus placebo, confirming that RVX-208 can modulate complement expression in humans. Although some factors were altered in both trials, several were identified in ASSERT but not ASSURE, and vice versa. This may be explained by differences in target population (stable versus unstable CAD) and trial duration (12 versus 26 weeks). In fact, patients enrolled in ASSURE displayed higher levels of CRP at baseline, indicating a higher inflammatory state as compared to patients from the ASSERT trial (Supplemental Fig. 7). Since complement expression and activation is affected by systemic low-grade inflammation in CVD patients [36, 37], and that inflammation-driven complement expression can be reduced by RVX-208 treatment, it is conceivable that RVX-208 could have differential effects in patients with dissimilar inflammatory profiles. For instance, in patients with unstable CAD enrolled in the ASSURE trial, RVX-208 lowered levels of cleaved complement fragments C5a (51%) and C5b-C6, a precursor to C5b-9 (10%). Interestingly, abundance of these fragments reflects systemic activation of the terminal complement pathway, which is prominent in advanced stages of CVD and during acute events [4]. Specifically, C5a correlates with secondary CVD events or restenosis in patients with coronary heart disease and peripheral artery disease [38–40], while circulating C5b-9 levels predict risk of death or functional outcome in patients with acute myocardial infarction and heart failure [41, 42]. Thus, downregulation of markers of complement activation by RVX-208 in patients with advanced CAD may offer therapeutic benefits with respect to CVD progress and MACE risk.

In patients with stable CAD enrolled in the ASSERT trial RVX-208 lowered levels of the cleaved complement peptide C3b (32%), again supporting the idea that RVX-208 modulates complement activation in vivo. C3b levels have not been previously correlated with CVD in clinical studies, but C3a and C3a-desArg, which are generated in the same proteolytic reaction, are linked to CVD risk factors both at early and late stages of the disease. Specifically, C3a is independently associated with markers of atherosclerosis [43] and predicts rehospitalization, cardiovascular events, and mortality [44]. C3a-desArg positively correlates with CVD risk factors such as obesity, blood pressure, blood lipids, and the metabolic syndrome [36]. In the same patient population RVX-208 reduced circulating levels of C3 and C4 (13 and 10%, respectively). Plasma concentrations of C3, C4, and the C4/C3 ratio have been linked to classic CVD risk factors, low-grade inflammation, and the metabolic syndrome [4, 36, 37, 43, 45, 46]. Studies have shown that even a small reduction in circulating C3 and C4 levels can offer protection against MACE [36, 46–48], making modulation of circulating complement factors an attractive pharmaceutical target.

CRP and APCS are liver-derived, clinically relevant inflammatory markers from the pentraxin family that activate the complement system via the C1q complex [49]. Inflammatory expression of CRP and APCS was downregulated by RVX-208 in PHH (Fig. 4e, f). Circulating CRP levels in RVX-208-treated patients were also reduced by 45% (*p* = 0.01) in ASSERT and by 52% (*p* = 0.0001) in ASSURE, as compared to placebo. Further, APCS was reduced by 11% (*p* = 0.001) in ASSERT patients. Although CRP and APCS are not the sole complement activators, their decrease in RVX-208-treated patients may be linked to lower levels of downstream cleavage products. As an upstream activator of the complement cascade CRP correlates with circulating complement anaphylatoxins C3a and C5a [44]. Conversely, during inflammatory response C5a acts in concert with IL-6 and/or IL-1β to promote upregulation of the CRP and APCS genes [50]. Thus, it is possible that RVX-208 disrupts this signaling by downregulating CRP and APCS expression, which could prevent amplification of activity of the classical complement pathway during chronic inflammation.

In addition to pentraxins, multiple other activators of the complement cascade were downregulated by RVX-208 in clinical samples including collectin-11, collectin-12, and properdin. Cascade inhibitors complement factor I, CD55, C1INH, and vitronectin were also reduced. Considering that complement activators and inhibitors form a complex network of interactions that involves multiple cell types [1], the net effect of RVX-208 on complement cascade activation in vivo likely depends on the biological context. Nonetheless, lower circulating levels of cleaved C3 and C5 fragments in RVX-208-treated patients indicate that, at least in plasma, RVX-208 tilts the balance towards a decrease in complement cascade activity and potentially in anaphylatoxin-driven inflammation.

Consistent with the widespread effect of RVX-208 on complement levels in the ASSURE trial, the complement system was ranked by IPA® as the second canonical pathway out of 351 significantly affected by RVX-208 treatment in patients’ plasma (*p* = 0.01; data not shown). This observation is largely in agreement with gene microarray data from both PHH donors where the complement system was also ranked as a top canonical pathway downregulated by RVX-208 (Fig. 1a). These findings point to a broad and coordinated downregulation of the cascade components and regulators at both the mRNA and protein level, potentially leading to a new activation state of the complement cascade.

The homeostasis of inflammatory responses is crucial for maintenance of the health balance. Just as overactive complement can be damaging to host tissue, complete inhibition of complement activity may interfere with its immune function [51]. Importantly, treatment with RVX-208 coordinately downregulates expression of complement factors in patients without any evidence of increased infections or infestations (Supplemental Table 1). This suggests that RVX-208 reduces both the levels and pro-inflammatory activity of complement without impairing the immune function of the cascade. Thus, in addition to improving the lipoprotein and inflammatory profile [10], RVX-208 may benefit CVD patients by modulating the complement cascade. In summary, inhibition of BET proteins by RVX-208 offers a multifactorial therapeutic strategy which may improve CVD and decrease MACE risk. Ongoing clinical studies will shed light on the safety and efficacy of RVX-208 in CVD patients [52].

## Electronic supplementary material

ESM 1 (PDF 204 kb)

ESM 2 (PDF 271 kb)

## Abbreviations

APCS: Amyloid P component serum
BET: Bromodomain and extraterminal
BETi: BET inhibitor
C4BP: C4b-binding protein
CAD: Coronary artery disease
CVD: Cardiovascular disease
CPN: Carboxipeptidase N
CRP: C-reactive protein
FB: Factor B
FD: Factor D
HDL: High-density lipoprotein
FH: Factor H
FI: Factor I
IL-1: Interleukin-1
IL-6: Interleukin-6
INFγ: Interferon γ
MACE: Major adverse cardiac event
MASPs: MBL-associated serine proteases
MBL: Mannose-binding lectin
PHH: Primary human hepatocytes
PRRs: Pattern recognition receptors
TNFα: Tumor necrosis factor α
