# Supplementary material for: Downregulation of the Complement Cascade In Vitro, in Mice and in Patients with Cardiovascular Disease by the BET Protein Inhibitor Apabetalone (RVX-208)
Source: J Cardiovasc Transl Res. 2017 May 31;10(4):337–47. doi: 10.1007/s12265-017-9755-z (PMC5585290; doi:10.1007/s12265-017-9755-z)
Supplement: Supplementary file 1 — (PDF 204 kb) [file 12265_2017_9755_MOESM1_ESM.pdf]

Wasiak et al. Supplemental material

Supplemental Figure Legends

Supplemental Fig. 1 RVX-208 downregulates expression of C4, C5 and C9 in primary human hepatocytes in a dose-dependent and time-dependent manner

a PHH were treated with RVX-208 for 72h. Gene expression was analyzed by real time PCR and expressed relative to vehicle (1). **b** PHH were treated with 30μM RVX-208 for 3h, 24h, 48h and 72h. Gene expression was analyzed by real time PCR and expressed relative to vehicle (1). Standard deviation was calculated on technical triplicates. Representative data is shown.

Supplemental Fig. 2 BETi downregulate mRNA expression and protein secretion of complement factors in Huh-7 cells

a Half maximal inhibitory concentration was determined for complement genes expressed in Huh-7 cells treated with RVX-208 and JQ1 for the indicated time. ND: not determined. **b** Complement secretion in response to increasing concentrations of RVX-208 (open symbols) or 0.3μM JQ1 (solid symbols) was analyzed by ELISA, normalized to secreted transferrin and expressed as relative to vehicle (1). Standard deviation was calculated on biological triplicates.

Supplemental Fig. 3 JQ1 downregulates expression of complement components in primary human hepatocytes in a time-dependent manner

PHH were treated with 0.3μM JQ1 for 3h, 24h, 48h and 72h. Gene expression was analyzed by real time PCR and expressed as relative to vehicle (1). Standard deviation was calculated on technical triplicates. Representative data is shown.

Supplemental Fig. 4 RVX-208 reduces inflammatory complement expression in Huh-7 cells

Huh-7 cells were treated with 30μM RVX-208 in combination with IL-6, interferon γ, IL-1β or TNFα (10ng/mL) for a total of 72h. Gene expression **(a)** was analyzed by real time PCR and expressed as relative to vehicle. Protein secretion **(b)** over the final 24h of treatment was analyzed by ELISA, normalized to cell number and expressed as relative to vehicle (1). Standard deviation was calculated on technical triplicates. Representative data is shown. Student's t-test; * $0.05>p>0.01$; ** $0.01>p>0.001$; *** $p<0.001$.

Supplemental Fig. 5 RVX-208 reduces pre-established inflammatory complement expression in PHH

PHH were pre-treated with IL-6 **(a-b)** or interferon γ **(c-d)** (10ng/mL) for 24h before 30μM RVX-208 was added to tissue culture media for the final 48h. Gene expression **(a and c)** was analyzed by real time PCR at 72h and expressed as relative to vehicle. Protein secretion **(b and d)** over the final 24h of treatment was analyzed by ELISA, normalized to cell number and expressed as relative to vehicle (1). Standard deviation was calculated on technical triplicates. Representative data is shown. Student's t-test; * $0.05>p>0.01$; ** $0.01>p>0.001$; *** $p<0.001$.

Supplemental Fig. 6 RVX-208 reduces pre-established inflammatory complement expression in Huh-7 cells

Huh-7 cells were pre-treated with IL-6, interferon γ, IL-1β or TNFα (10ng/mL) for 24h before 30μM RVX-208 was added to tissue culture media for the final 48h. Gene expression after 72h **(a)** was analyzed by real time PCR and expressed as relative to vehicle. Protein secretion **(b)** over the final 24h of treatment was analyzed by ELISA, normalized to cell number and expressed as relative to vehicle (1). Standard deviation was calculated on technical triplicates. Representative data is shown. Student's t-test; * 0.05>p>0.01; **0.01>p>0.001; *** p<0.001.

Supplemental Fig. 7 Levels of the C-reactive protein in baseline plasma samples from phase 2b clinical trials ASSERT and ASSURE*

*Mann-Witney test between the two groups

Supplemental Table Legends:

Supplemental Table 1 Incidence of infections and infestations in the ASSERT and ASSURE clinical trials
