# Supplementary material for: Downregulation of the Complement Cascade In Vitro, in Mice and in Patients with Cardiovascular Disease by the BET Protein Inhibitor Apabetalone (RVX-208)
Source: J Cardiovasc Transl Res. 2017 May 31;10(4):337–47. doi: 10.1007/s12265-017-9755-z (PMC5585290; doi:10.1007/s12265-017-9755-z)
Supplement: Supplementary file 2 — (PDF 271 kb) [file 12265_2017_9755_MOESM2_ESM.pdf]

Supplemental Fig. 1 Wasiak et al.

a

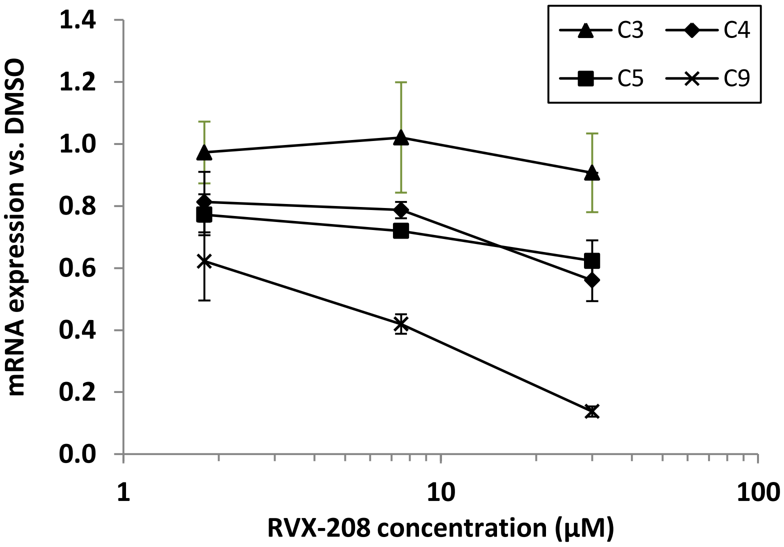

b

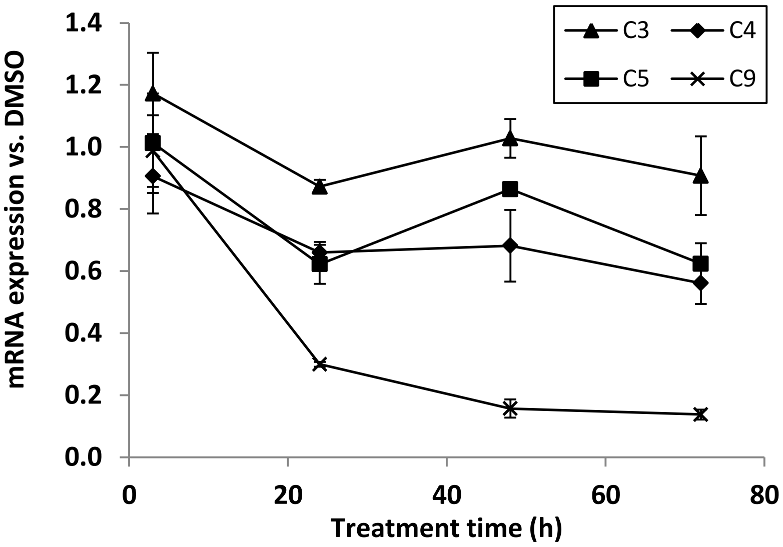

Supplemental Fig. 2 Wasiak et al.

a

	IC50 (uM)	
**Complement gene (Treatment time)**	**RVX-208**	**JQ1**
MBL2 (48h)	16.3	0.16
C1S (48h)	18.9	0.30
C2 (48h)	>50	ND
C3 (48h)	1.90	0.20
C4a/4b (48h)	6.30	ND
C5 (48h)	>30	ND
C5 (72h)	21.8	0.27

b

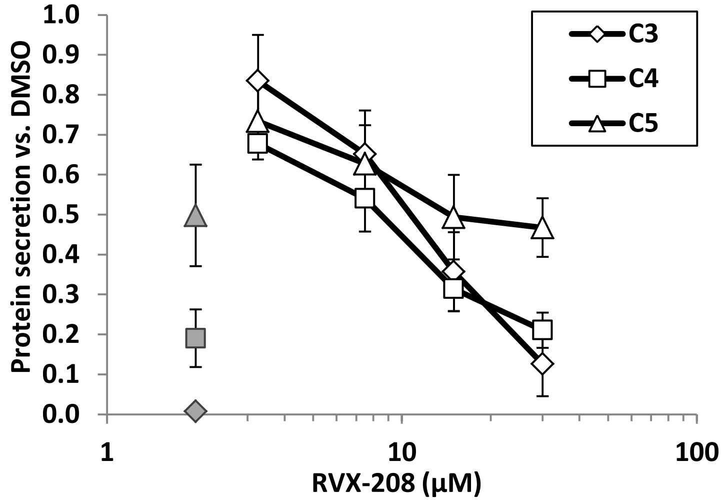

Supplemental Fig. 3 Wasiak et al.

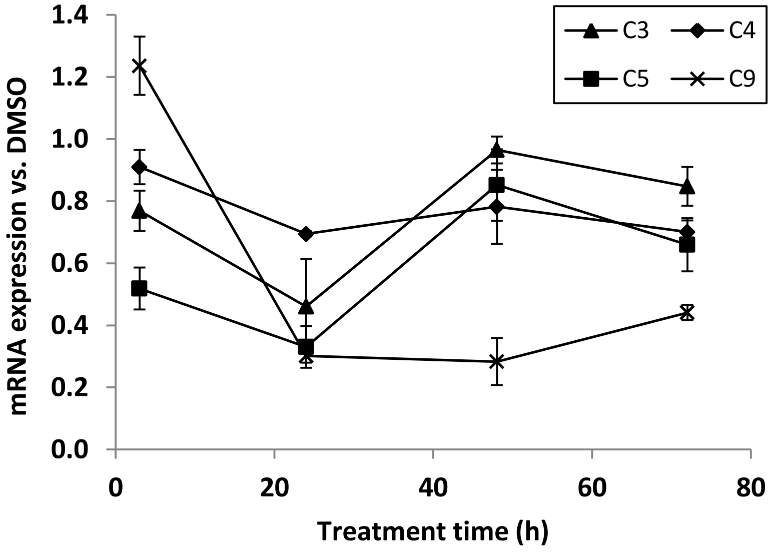

Supplemental Fig. 4 Wasiak et al.

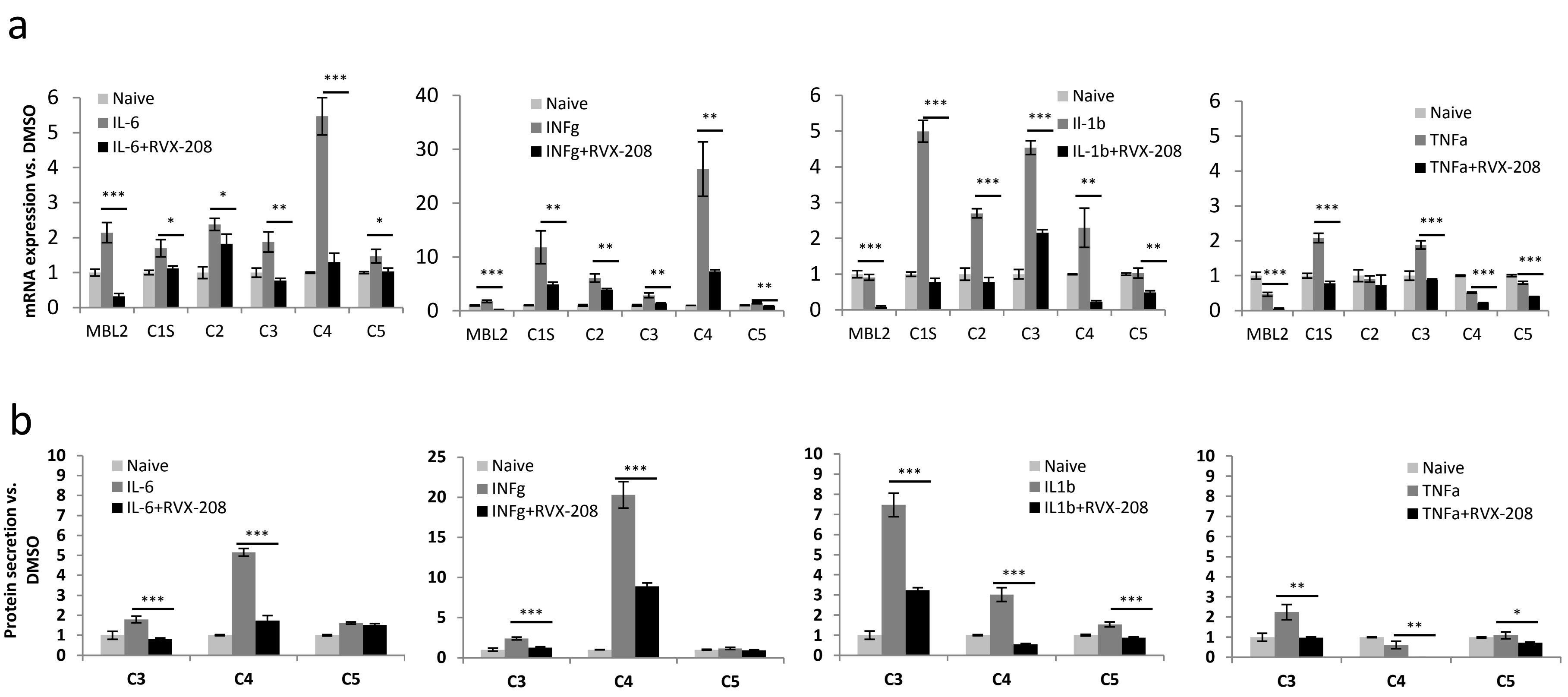

Supplemental Fig. 5 Wasiak et al.

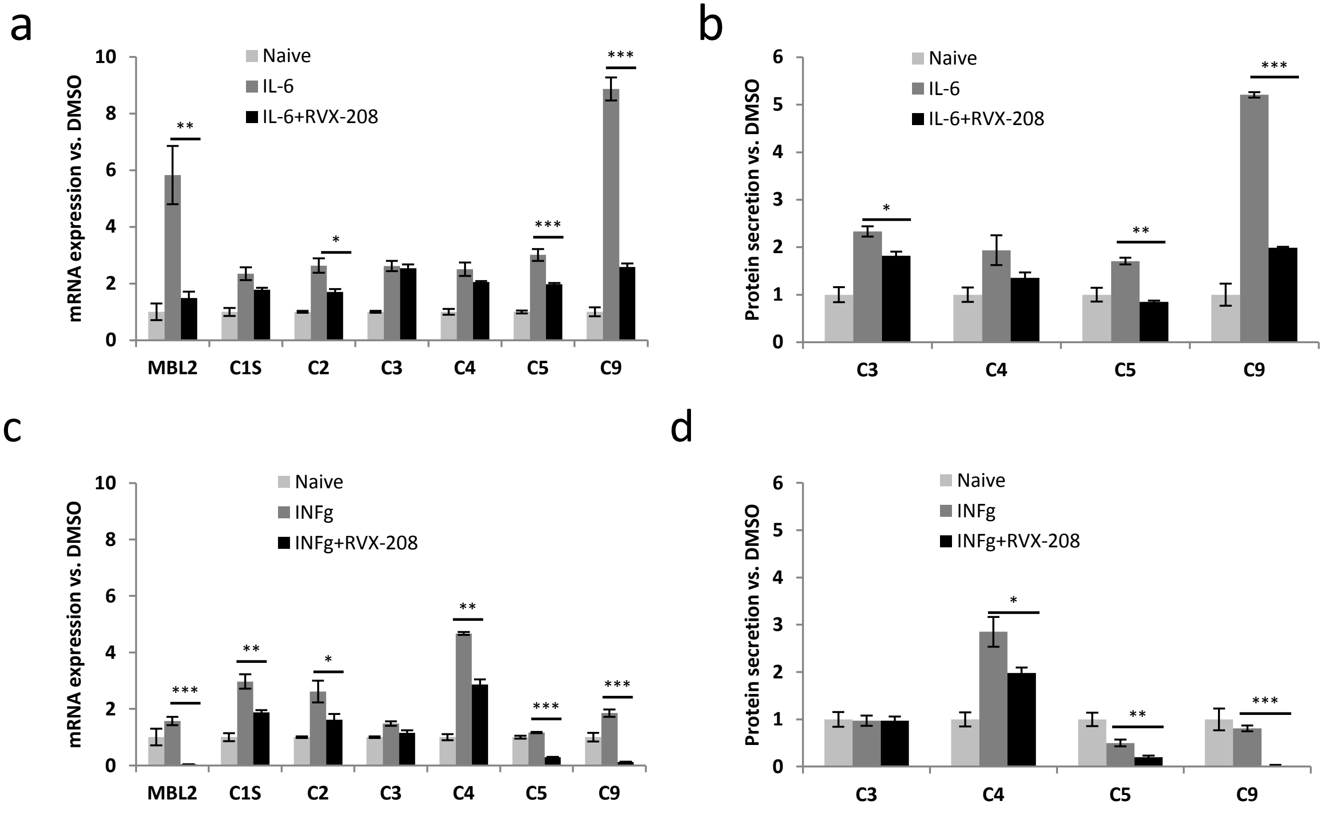

Supplemental Fig. 6 Wasiak et al.

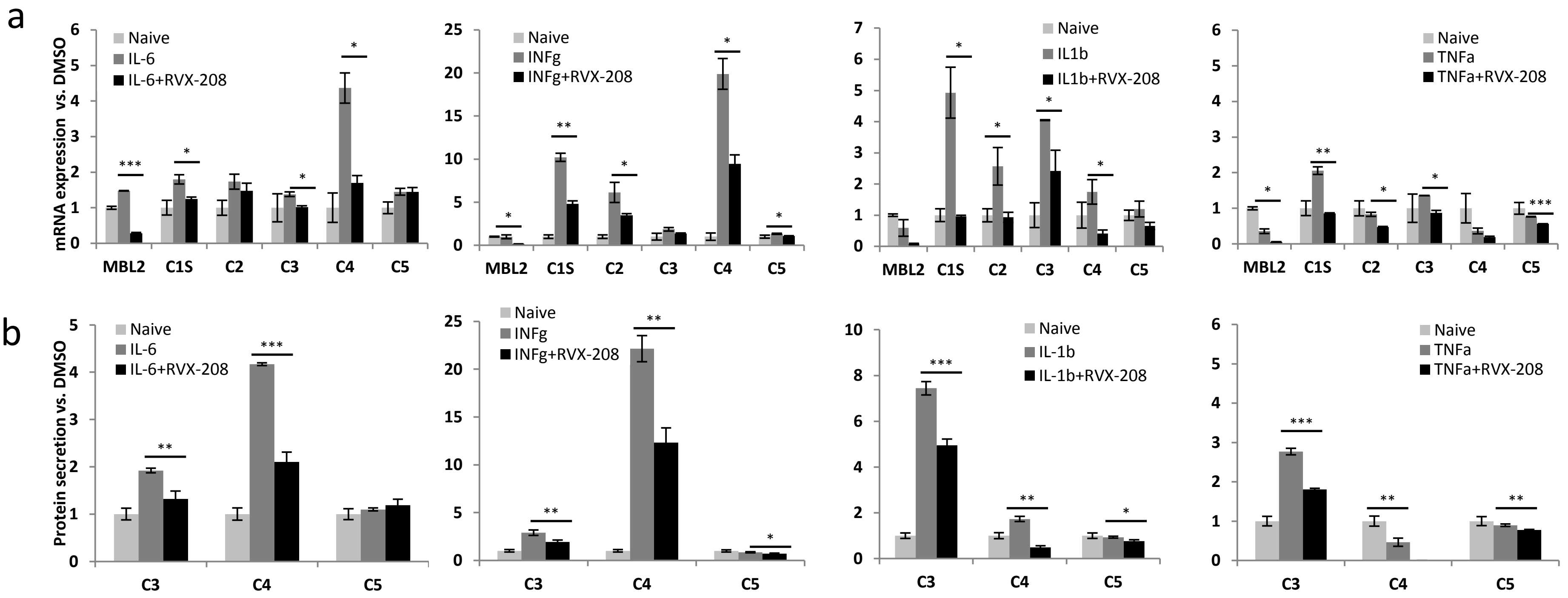

Supplemental Fig. 7 Wasiak et al.

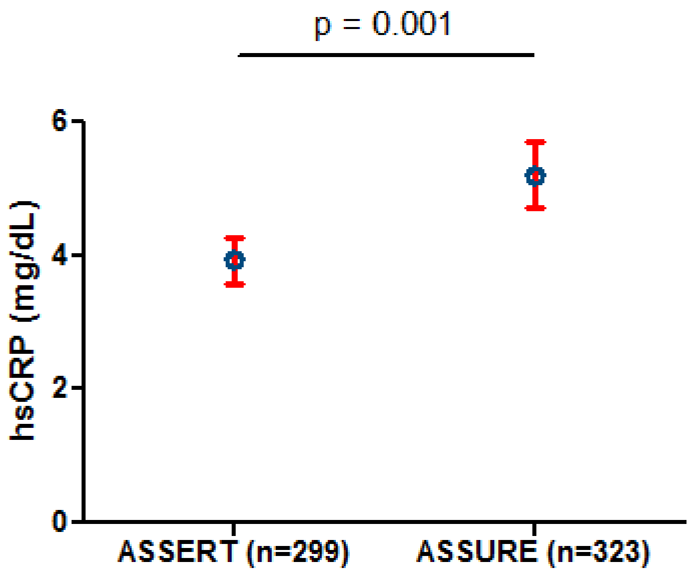

Supplemental Table 1 Wasiak et al.

Study	ASSERT 12-Week Study		ASSURE 26-Week Study		Total	
**Treatment**	**RVX-208 (N=75)**	**Placebo (N=74)**	**RVX-208 (N=243)**	**Placebo (N=80)**	**RVX-208 (N=318)**	**Placebo (N=154)**
**Infections and Infestations**	13 (17.3%)	16 (21.6%)	32 (13.2%)	10 (12.5%)	73 (15.6%)	26 (16.9%)
